# The correlation between serum bone metabolism indexes and bone disease and survival in newly diagnosed multiple myeloma patients

**DOI:** 10.1080/15384047.2024.2403205

**Published:** 2024-09-18

**Authors:** Linlin Huang, Yi Zhong, Qingxiao Chen, Donghua He, Gaofeng Zheng, Yang Yang, Xiaoyan Han, Wenjun Wu, Yi Zhao, Yi Li, Li Yang, Zhen Cai, Jingsong He

**Affiliations:** a Bone Marrow Transplantation Center, The First Affiliated Hospital, School of Medicine, Zhejiang University, Hangzhou, China; b Department of Hematology, The First Affiliated Hospital of Zhejiang Chinese Medical University (Zhejiang Provincial Hospital of Chinese Medicine), Hangzhou, Zhejiang, China

**Keywords:** Multiple myeloma, bone metabolism indexes, myeloma bone disease, β-CTX, PINP, N-MID

## Abstract

Objective Myeloma-related bone disease (MBD) is one of the most common complications of multiple myeloma (MM). This study aims to investigate the correlation between serum bone metabolism indexes (BMIs), the clinical characteristics and prognosis of newly diagnosed MM (NDMM) patients. Methods: The serum BMIs of 148 patients with NDMM in a single hematological disease treatment center from April 2014 to December 2019 were analyzed retrospectively, including type I collagen amino terminal elongation peptide (PINP), β-C-terminal telopeptide of type I collagen (β-CTX) and N-terminal osteocalcin (N-MID). Other clinical indexes were simultaneously collected and the degree of bone damage in patients was evaluated. We explored the effect of serum BMIs on the prognosis and identified independent prognostic factors. Another 77 NDMM patients from April 2018 to February 2021 served as the validation cohort. Results: The area under the curve (AUC) predicted by β-C-terminal telopeptide of type I collagen (β-CTX), type I collagen amino terminal elongation peptide (PINP), and N-terminal osteocalcin (N-MID) for overall survival (OS) were 0.708, 0.613, and 0.538, respectively. Patients with high serum levels had shorter OS (*p* < .001, *p* = .004, *p* = .027, respectively). Cox multivariate analysis indicated that serum β- CTX、lactic dehydrogenase、hemoglobin and the degree of bone injury were independent prognostic factors. A COX regression model was established with a C-index of 0.782 and validated with a C-index of 0.711. Conclusion: The serum BMIs are correlated with the patients’ OS, and β- CTX can be an independent prognostic factor.

Multiple myeloma (MM) is one of the common hematological malignant tumors. In recent years, its incidence has increased significantly in China. Myeloma-related bone disease (MBD) is one of the most common complications of MM patients, leading to progressive bone destruction, even pathological fractures, which not only significantly affects the quality of life of patients but also is one of the main causes of death.^1,2^ At least 80% of patients exhibit some degree of bone destruction at diagnosis, which is typically associated with tumor burden and prognosis.^3,4^ Serum bone metabolism indexes (BMIs) include N-terminal osteocalcin (N-MID), procollagen I amino terminal peptide (PINP) and β-C-terminal peptide of type I collagen (β-CTX) can reflect the activity of bone resorption and bone formation, thereby evaluating the bone metabolism status of MM patients,^5^ indicating the degree of bone destruction, and finding a certain correlation with the remission or progression of MM disease.^6^ However, there are few reports on whether the serum levels of the BMIs can indicate the survival prognosis of MM patients. This study analyzed the correlation between serum BMIs above and the degree of bone damage in newly diagnosed MM(NDMM) patients from 2014 to 2019 and explored their relationship with the prognosis of MM patients, in order to provide a basis for the application of serum BMIs to the assessment of the degree of bone damage and the prognosis of the disease in MM patients.

## Patients and methods

### Patients

This article retrospectively analyzed complete clinical data including serum samples and clinical data of 148 NDMM patients in a single hematological disease treatment center from April 2014 to December 2019. The external validation cohort consisted of 77 NDMM patients from April 2018 to February 2021. MM diagnosis and response assessment were defined by the International Myeloma Working Group’s criteria.^7,8^

### Clinical data

Serum BMIs including N-MID, PINP, and β-CTX detection kits (including calibration and quality control) were purchased from Roche, Germany, and were tested using a Roche fully automated electrochemical luminescence analyzer. Clinical routine tests are used for serum creatinine (Cr), hemoglobin (Hb), serum calcium and lactate dehydrogenase (LDH), etc. Fluorescence in situ hybridization (FISH) technology was used to detect specific genetic abnormalities in patient bone marrow MM cells, including 1q amplification, 13q deletion, 17p deletion, and IGH rearrangement, with a cutoff value of 20% for deletion and amplification and 10% for IGH rearrangement.^9^ The above information was retrospectively obtained from the patient medical record system. Imaging (X-ray, CT, MRI, PET-CT) was used to determine whether a patient has bone destruction and the extent of the destruction. PET-CT was the first choice, and CT and MRI were the second choice. The degree of bone damage in patients was divided into six parts according to their location, respectively, the skull, scapula, vertebrae, sternum, ribs, clavicle, ilium, pelvic bone, sacrum, pubis femur, and humerus. Each part must have at least one bone destruction greater than 5 mm, with a score of 1 point, and then the score of bone damage in patients was calculated.^10^

### Follow up

The follow-up data was collected from medical records, and telephone follow-up records. The deadline for follow-up was June 30, 2020 and October 31, 2022 for the external validation queue. Overall survival (OS) was defined as the time from the beginning of treatment to death from any cause or the last follow-up, while progression free survival (PFS) was defined as the time from the beginning of treatment to disease progression or death or the last follow-up.

### Statistical analysis

IBM SPSS 23.0 and R software 4.1.2 were used for plotting and statistical analysis. The Kolmogorov Smirnov method was used to test the normality of quantitative data, and descriptive quantitative data was represented by median/mean. Qualitative data was expressed as a percentage, and Chi-square test was used for comparing categorical data. The t-test and Wilcoxon test were used for comparing data between two groups. R (timeROC package) was used to draw ROC curves and calculate AUC; R (survminer package) was used to calculate the cutoff values of BMIs and common clinical indicators of patients and determine their high-level and low-level grouping. Survival curves were plotted using the Kaplan-Meier method, and survival rates were compared using the Log-rank test. Cox regression was used for univariate and multivariate analysis. A P-value less than 0.1 was used for multivariate analysis, with hazard ratio (HR) and 95% confidence interval (95% CI) displayed. Based on the results of multiple factor regression, we established and tested a nomogram, using Bootstrap resampling method to validate the model and calculating the C index. *p* < .05 was considered statistically significant.

## Results

### Clinical characteristics of patients

The median age of 148 patients was 64 (range: 56–69) years old, of which 84 were males (56.76%). According to the International Staging System (ISS),^11^ there were 55 cases (37.16%) in Stage I, 38 cases (25.68%) in Stage II, and 55 cases (37.16%) in Stage III. Among them, 126 patients underwent FISH testing at initial diagnosis, and 63.49% of patients had cytogenetic abnormalities. The positive rates for 1q gain or amplification, 13q deletion, IGH rearrangement, and 17p deletion were 48.41%, 34.92%, 30.95%, and 6.34%, respectively. The clinical characteristics of the patients are detailed in Table 1. All patients received first-line treatment with bortezomib- based regimen,^12^ including bortezomib combined with dexamethasone (Vd regimen), cyclophosphamide (VCd regimen), doxorubicin (VAd regimen), and thalidomide (VTd regimen) or lenalidomide (RVd regimen) on the basis of Vd regimen. Some patients over 70 years old with comorbidities or unfitness received weekly treatment with bortezomib. After receiving 6 courses of induction therapy, nineteen patients were given autologous hematopoietic stem cell transplantation (ASCT) based on the efficacy and patient’s wishes. The median number of induction treatment courses received by all patients was 3 (2–8). The detailed information of all patients, including the external validation set, is listed in Table 1. And 77 patients from April 2018 to February 2021 who had their BMIs measured at the time of initial diagnosis were included as validation cohort. The diagnostic criteria and laboratory index detection methods were consistent with those of the experimental cohort. The baseline characteristics of the validation cohort are listed in appendix (Table S1). All patients received bisphosphate therapy according to the diagnostic and treatment guidelines, and intravenous zoledronic acid injection or oral alendronate sodium were chosen based on the patient’s renal function.

**Table 1.** Clinical characteristics of 148 MM patients.

Clinical characteristics	*N* = 148
Age, median (range)	64(56-69)
Male, n (%)	84(56.76)
ISS, n (%)	
I	55(37.16)
II	38(25.68)
III	55(37.16)
M-protein, n (%)	
IgG	64(43.24)
IgA	41(27.70)
IgD	6(4.05)
Light chains only	36(24.32)
Biclonal subtype	1(0.68)
Bone destruction, n(%)	106(71.62)
Extramedullary infiltration, n(%)	38(25.68)
Serum creatinine (μmol/L), median (Qua)	79.00(63.00-126.00)
Serum calcium (mmol/L), median (Qua)	2.21(2.04-2.39)
Hemoglobin (g/L), median (Qua)	96.50(76.00-111.50)
Platelet count (10^9/L), median (Qua)	185（118.50-228.25）
Lactate dehydrogenase (U/L), median (Qua)	190.50(153.50-237.25)
β2-microglobulin (mg/L)，median (Qua)	3570.00(1975.00-6825.00)
Albumin (g/L)，median (Qua)	38.55(34.40-44.30)
Cytogenetic abnormalities ^a^(%)	63.49(80/126)
High risk ^b^(%)	50.79(64/126)
Standard risk(%)	3.17(4/126)
Unknown ^c^(%)	9.52(12/126)
Induction therapy, n(%)	
Vd	26(17.57)
VCd	78(52.70)
VAd	24(16.22)
VTd/RVd	18(12.16)
ASCT received, n(%)	19(12.84)
Best response, n(%)	
CR	73(49.32)
VGPR	29(19.59)
PR	36(24.32)
<PR	10(6.76)

ISS: International Staging System; a: cytogenetic data from 126 cases were available; b: High-risk status was determined as having 1q amplification or 17p deletion according to mSMART 3.0; c: IGH rearrangement only, or both IGH rearrangement and 13q deletion were included in the unknown group; **Q**ua: quartile; PD: bortezomib, dexamethasone; PCD: bortezomib, cyclophosphamide, dexamethasone; PAD: bortezomib, doxorubicin, dexamethasone; PTD: bortezomib, thalidomide, dexamethasone; CR: complete remission; VGPR: very good partial remission; PR: partial remission.

### Serum BMIs, ROC curves, and cutoff values

**Figure 1.**
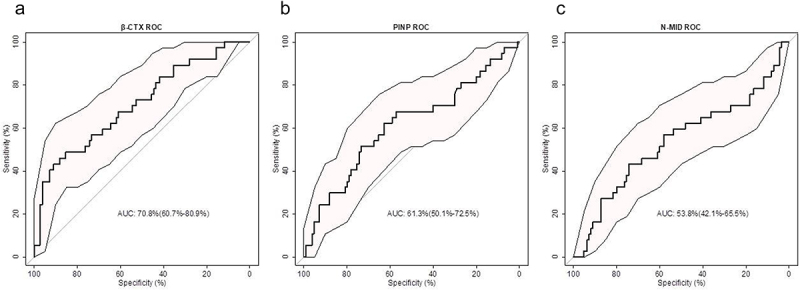
ROC curves for the predictive value of various serum bone metabolism indexes in MM patients’ prognosis: β-ctx(a), PINP(b), N-MID(c) and β-ctx/pinp (d).

Serum levels of β-CTX, PINP, and N-MID were 768.5 μg/ml (range:468.95-1243.50 μg/ml), 49.95 ng/ml (range:34.23-74.75 ng/ml), 22.53 ng/ml (range:15.34-35.67 ng/ml), respectively. The ROC curve was used to analyze the predictive effect of various serum BMIs on patients’ OS (Figure 1). “The areas under the curve (AUCs) for predicting OS in MM patients were 0.708 (95% CI: 0.607–0.809) for β-CTX, 0.613 (95% CI: 0.501–0.725) for PINP, and 0.538 (95% CI: 0.421–0.655) for N-MID. The cut-off values for β-CTX, PINP, and N-MID were 1304.00 μg/ml, 65.55 ng/ml, 32.00 ng/ml, respectively. Those higher than the above values were defined as high-level groups, while those below were defined as low-level groups.

β-CTX: β-C-terminal telopeptide of type I collagen; PINP: type I collagen amino terminal elongation peptide; N-MID: N-terminal osteocalcin

### The correlation between serum levels of BMIs and the degree of bone destruction in patients

According to the imaging results of patients, the locations of bone damage were counted, and then the correlation between the serum levels of various BMIs and the number of bone damage sites was analyzed. A bone damage score greater than 3 was classified as a severe damage group. The results showed that the serum level of β-CTX was correlated with the degree of bone damage. 22.58% (7/31) of patients with high serum levels of β-CTX had severe bone damage, while only 9.40% (11/117) of patients with low serum levels had severe bone damage. No correlation was found between serum PINP and N-MID levels and the degree of bone lesions in patients (Table 2).

**Table 2.** Correlation analysis of serum BIMs level and the degree of bone lesions in patients.

Serum BIMs	N	Bone destruction (>3),n(%)	Bone destruction (≤3分),n(%)	*Χ*^*2*^	*P*
β-CTX				3.984	0.046
>1304(μg/ml)	31	7 (22.58)	24 (77.41)		
≤1304(μg/ml)	117	11 (9.40)	106 (90.60)		
PINP				0.203	0.653
>66(ng/ml)	48	5 (10.42)	43 (89.58)		
≤66(ng/ml)	100	13 (13.00)	87 (87.00)		
N-MID				0.083	0.773
>32(ng/ml)	45	6 (13.33)	39 (86.67)		
≤32(ng/ml)	103	12 (11.65)	91 (88.35)		

### Correlation analysis between serum BMIs levels and other clinical characteristics

R language (cut off package) was used to calculate the cutoff values of various clinical indicators and round them up, which were LDH (246 U/L), Cr (140 μmol/L), Hb (79 g/L), platelet count (Plt, 140 × 109/L), β2-Microglobulin (β2-MG, 6420 mg/L) and albumin (41.6 g/L). Based on the cutoff values, the above clinical indicators were divided into high-level and low-level groups. The levels of serum BMIs were analyzed between different groups of the above clinical indicators. The results showed that there was a positive correlation between Hb, serum LDH, β2-MG, Cr levels and serum β-CTX level (*p* < .01), as well as ISS staging, while higher-risk ISS staging had higher serum β-CTX level (Table 3). PINP level showed a positive correlation with serum Cr and β2-MG levels (*p* < .05). N-MID also correlated positively with serum Cr, Hb, and β2-MG levels (*p* < .01). In addition, a total of 126 patients underwent FISH testing at initial diagnosis. It was shown that patients with 1q copy number > 2 had lower PINP values than those without 1q copy number increase (median: 49.13 ng/mL vs 54.85 ng/ml, *p* = .067), but there was no statistical significance (Table 3). There was no significant correlation between the levels of other serum BMIs and MM cytogenetic abnormalities.

**Table 3.** Correlation analysis of serum BIMs level and other clinical indexes in patients.

	β-CTX（*P* value）	PINP（*P* value）	N-MID（*P* value）
LDH, low vs high^†^	.004	.151	.738
Serum creatinine, low vs high	<.001	<.001	<.001
Hb, low vs high	<.001	.427	.004
Platelet count, low vs high	.293	.069	.418
β2-microglobulin, low vs high	<.001	.041	.002
Albumin, low vs high	.973	.244	.672
Cytogenetic abnormalities			
1q amplification, negative vs positive	.918	.067	.868
17p deletion, negative vs positive	.760	.730	.512
13q deletion, negative vs positive	.596	.802	.307
IGH rearrangement, negative vs positive	.757	.417	.591
D-S stage			
I vs II	.879	.744	.283
II vs III	.121	.243	.589
I vs III	.133	.699	.771
ISS			
I vs II	.350	.609	.479
II vs III	.002	.185	.058
I vs III	<.001	.068	.003

LDH: lactate dehydrogenase; Hb: hemoglobin; D-S: Durie-Salmon staging.

†low vs high: The above clinical indicators (LDH、serum creatinine、Hb、platelet count、β2-microglobulin、albumin) were divided into high-level groups (high) and low-level groups (low) based on the cutoff values.

### Survival analysis based on serum BMIs levels

**Figure 2.**
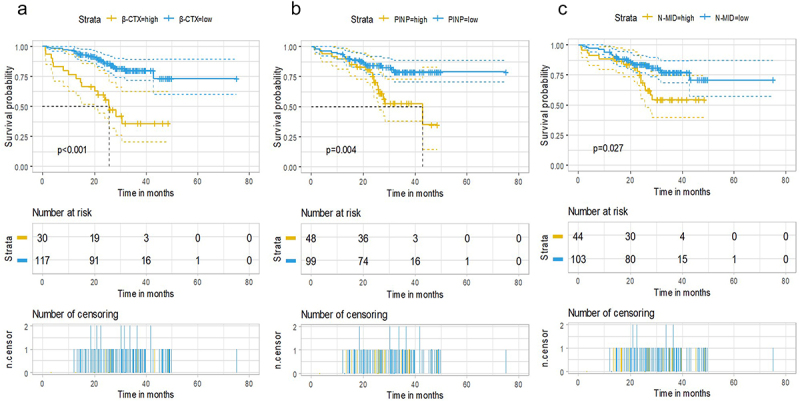
Overall survival curves of high/low serum BMIs level groups: β-CTX(2A), PINP(2B), N-MID(2C).

The median follow-up time of 148 patients was 31.7 m, with a median PFS of 38.7 m (95% CI: 23.3 m-NR). The 3-year and 5-year PFS were 52.3% and 38.6%, respectively. The median OS has not reached (NR) yet, with a 3-year and 5-year OS of 70.6% and 65.9%, respectively. BMIs were used to divide patients into high-level and low-level groups based on the cutoff values and then we plotted survival curves and used log-rank tests. The results showed that high serum levels of β-CTX, PINP and N-MID were significantly correlated with inferior OS (the median OS: 25.67 m vs NR, *p* < .001; 42.87 m vs NR, *p* = .004; NR vs NR, *p* = .027, respectively) (Figure 2).

### Cox regression analysis

The degree of bone damage, Hb, platelet count, serum BMIs (β-CTX, PINP, N-MID), Cr, LDH, β2-MG, ISS staging and cytogenetic abnormalities were included in the Cox model for univariate analysis, and variables were selected for multivariate analysis. The results showed that the degree of bone damage, Hb, serum LDH and β-CTX were independent risk factors affecting the OS of MM patients (Table 4).

**Table 4.** Cox regression analysis of risk factors affecting OS.

Variables	univariate analysis		multivariate analysis	
	*HR(95%CI)*	*P*	*HR(95%CI)*	*P*
Serum creatinine	2.76(1.39-5.47)	.004	1.16(0.41-3.31)	.782
Hb	0.28(0.14-0.53)	<.001	0.37(0.17-0.79)	.011
LDH	4.40(2.28-8.48)	<.001	4.52(2.16-9.47)	<.001
β2-microglobulin	2.90(1.50-5.63)	.002	1.10(0.39-3.06)	.858
Albumin	0.51(0.24-1.09)	.081	0.53(0.23-1.23)	.139
N-MID	2.05(1.07-3.94)	.031	0.79(0.32-1.93)	.609
β-CTX	4.23(2.21-8.10)	<.001	2.35(1.02-5.43)	.046
PINP	2.52(1.31-4.82)	.005	1.47(0.55-3.94)	.443
Bone destruction	2.04(0.93-4.48)	.075	2.80(1.13-6.94)	.027

### Nomogram predicts survival outcomes

**Figure 3.**
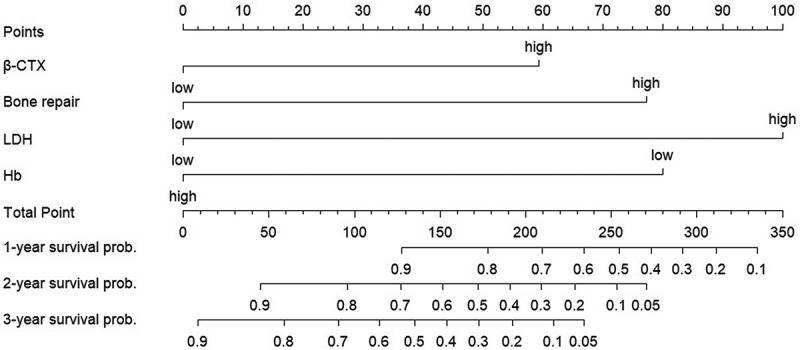
Nomograms for predicting OS.

**Figure 4.**
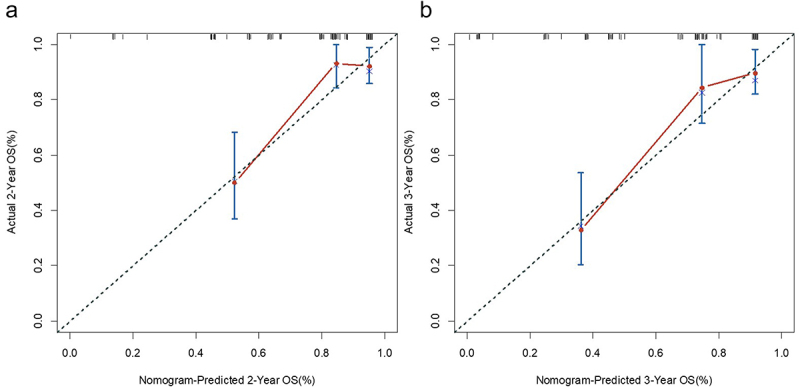
The calibration curves for predicting OS at 2-year (4A) and 3-year (4B).

In this study, a nomogram for predicting OS was constructed based on β-CTX, LDH, Hb, and the degree of bone damage (Figure 3). The Bootstrap resampling method was used to validate the nomogram, and a correction curve was drawn (Figure 4), with a C-index of 0.782 (95% CI: 0.698–0.866). The nomogram of this study suggests that the model based on the above four parameters has good predictive ability for patients’ OS. Based on the four indicators mentioned above, the prognosis model was validated using the external validation set, with a C-index of 0.711. To visually display the stratification power, Kaplan Meier analysis was performed to evaluate survival outcomes (Figure S1). Patients were stratified into two risk groups, and the cutoff score was value was 177.338. The results showed that high risk groups were significantly correlated with inferior OS (*p* < .001).

## Discussion

β-CTX and PINP are both sensitive biomarkers of bone metabolism, as recommended by the International Osteoporosis Foundation (IOF) and the International Federation of Clinical Chemistry (IFCC). PINP is a type I procollagen peptide, an organic component of bone, cleaved by a specific protease, and is an indicator of collagen synthesis. Therefore, it is a specific marker that reflects bone formation. β-CTX is a fragment product of mature type I collagen degradation, which is a specific indicator of bone resorption function.^13–15^ N-MID is an important bone specific calcium binding protein in the bone matrix, produced by osteoblasts and closely related to the bone turnover rate of bone metabolic diseases.^16^ Normally, the above indicators represent physiological bone turnover, but in pathological conditions, when the balance between bone resorption and osteogenesis is disrupted, these serum indicators will undergo changes.^17^

Bone destruction is one of the most important clinical features of MM patients, which can also lead to abnormal bone turnover indicators. Wang et al.^18^ compared BMIs between 55 patients with MBD and 20 healthy individuals. The results showed that serum β-CTX level and the ratio of β-CTX to PINP were significantly higher in the MBD group than in the healthy control group (*p* < .01), which meant β- CTX could effectively reflect the degree of bone damage in MM patients. In an analysis of MM patients with different grades of bone disease, the group with more severe bone disease grading had significantly higher levels of β-CTX and tPINP, and β-CTX/tPINP ratio (all *p* < .05) which could be used to assess the severity of myeloma-associated bone disease at diagnosis.^19^ The results of Yang Shiwei et al.^20^ also showed that the β-CTX/PINP ratio was higher in MM patients than in healthy control (*p* = .001), and positively correlated with the severity of MBD, while the levels of β-CTX and PINP were not statistically significant in monitoring the severity of bone disease. Our study included a larger number of cases, totaling 148 newly diagnosed MM patients, and tested their serum β- CTX, PINP, N-MID levels before treatment. Consistent with previous findings, a significant positive correlation was found between high serum levels of β-CTX and more severe bone disease, and serum BMIs can be used as one of the evaluation indicators for bone destruction in MM patients, which can detect high-risk populations of bone destruction earlier than traditional imaging examinations.^2^ However, we did not find a correlation between PINP, N-MID serum levels and the degree of bone damage in patients, and further case studies may be needed.

In a cross-sectional study by Sonia Vallet et al.^21^ serum PINP and β- CTX levels were significantly higher than those of patients with monoclonal immunoglobulin disease with unknown significance (MGUS). In addition, PINP and β-CTX levels increased, but remained basically unchanged in stable MGUS patients. These data indicated the potential roles of PINP and β-CTX as biomarkers for MGUS progression to MM.^21^ The results of Liu et al.^6^ indicated that BMIs in MM patients had different levels in the stable and progressive stages of MM, with the latter having higher serum PINP and β-CTX levels. There are also studies that have not found a correlation between BMIs and disease progression, but they may suggest therapeutic effects, especially effective control of bone destruction.^18^ Previous studies have confirmed that the levels of tPINP, β-CTX and β-CTX/tPINP ratio were significantly decreased in patients with MBD when treatment was effective.^19^

A retrospective analysis of 106 patients with refractory and recurrent MM was conducted, while 99 patients were prospectively included.^22^ The results showed that bone resorption marker β-CTX decreased compared to before in patients who responded to the combination of lenalidomide and dexamethasone (RD) regimen, but bone formation indicators were not affected. In patients treated effectively with bortezomib-based regimens, bone resorption indicators decrease firstly, and bone formation indicators increase after 6 courses treatment. Therefore, it was believed that bortezomib may have a promoting effect on bone formation, while lenalidomide did not. It was recommended to use bortezomib for treatment in patients with obvious MBD. One study suggests that bortezomib inhibited osteoclast function, reduced bone resorption markers and normalized the RANKL/osteoprotegerin ratio in MM patients, while increasing osteoblast differentiation and activity, elevating bone formation indicators, and decreasing dickkopf-1 protein levels.^23^ In a Phase 2 study of transplant-eligible NDMM patients, RAD induction regimen (lenalidomide, adriamycin and dexamethasone) significantly reduced CTX and tartrate resistant acid phosphatase 5b (TRACP-5b) and also increased bone alkaline phosphatase (BALP), PINP and osteocalcin. It showed that RAD could improve bone metabolism.^24^ The results of Terpos E et al.^25^ showed that a lenalidomide-based regimen favorably affected bone metabolism in RRMM patients. In addition, ASCT may normalize abnormal bone resorption in MM patients by decreasing the RANKL/OPG ratio, while bone formation took longer to normalize.^26^ In a prospective phase 2 study of 33 patients treated with daratumumab, dickkopf-1 and C-C motif ligand-3 decreased significantly, while the levels of osteocalcin, BALP and PINP increased. Therefore, daratumumab may improve bone turnover by controlling osteoblast inhibition and inducing bone formation.^27^ Mara Kowalska et al.^28^ also believed that the bone formation marker PINP may have important significance in monitoring the response after drug therapy with combined anti-myeloma activity and bone protective activity. Therefore, BMIs may become an important factor indicating efficacy in the treatment process of MM patients.

However, there is little research on the correlation between serum BMIs and the prognosis of MM patients at initial diagnosis. A recent study^29^ analyzed the correlation between BMIs and MM cytogenetic abnormalities, and the results showed that patients with 1q amplification were associated with lower PINP level (*p* = .03), suggesting that 1q amplification may be involved in the development of MBD, but the specific mechanism still needs further research. Our results also revealed a certain correlation between 1q amplification and lower serum PINP level in patients, but there was no statistical significance (*p* = .067), and a larger sample may be needed for exploration. In the treatment of MBD, β-CTX has shown promise in detecting bone metastases, evaluating prognosis, and monitoring therapy,^30,31^ but Chu Bin et al.^19^ found no significant differences in median overall survival and median progress-free survival in MM patients with different grades of bone disease. Therefore, we further analyzed the correlation between the levels of serum BMIs and patients’ OS. In univariate analysis, it was found that high levels of β-CTX, PINP, N-MID were associated with shorter survival in newly diagnosed MM patients, especially β- CTX which was an independent prognostic factor for patient OS. And we discovered that β- CTX and serum LDH、β2-MG levels were positively correlated, showing that BMIs may indicate tumor burden in patients.

It was reported^6^ that the levels of PINP and β-CTX were significantly higher in the MM progression period than in the remission period. The mechanism may be related to the interaction between MM cells and osteoclasts/osteoblasts. MM cells can inhibit the differentiation of osteoblasts by secreting cytokines such as DKK1. At the same time, urinary amino-terminal cross-linking telopeptide of type I collagen (NTX) and serum CTX generated by matrix metalloproteinases (ICTP) elevated in MM patients with osteolytic lesions and correlated with disease progression and overall survival.^32^ MM cells and their stromal cells upregulate the expression of RANKL, inducing myeloid precursor cells to differentiate into osteoclasts, enhancing the function and quantity of osteoclasts, and thereby exacerbating the occurrence and development of osteolytic lesions in patients. On the other hand, osteoclasts also release factors that stimulate the growth of MM cells while dissolving bones, promoting the progression of MM disease.^33,34^ Therefore, patients with higher levels of BMIs may have higher tumor burden and poorer prognosis. The nomogram based on COX regression results in this study has good predictive ability. At the initial diagnosis, the serum BMIs of MM patients, especially β-CTX can be included as an important prognostic factor in the prognosis evaluation system of MM patients. However, it still needs to be verified and confirmed in more case cohorts, and its specific mechanism urgently needs to be further explored.

This study is a retrospective study with a limited number of cases, which to some extent affected the results. In addition to β-CTX, PINP, and N-MID, there are other indicators that reflect bone synthesis and bone resorption, such as tartrate resistant acid phosphatase 5b (TRACP-5b) which reflect bone resorption, BALP, and osteoprotegerin (OPG), which are markers of bone synthesis. In recent years, flow cytometry has also been used to detect osteoclast precursors (OCPs) and osteoblast precursors (OBPs) in patients’ blood circulation, which are more sensitive than traditional bone turnover markers.^35^ By further monitoring and analyzing these indicators, we can further explore their relationship with tumor progression and the occurrence and development of bone diseases. In addition, cytogenetic abnormalities in MM patients are often related to their clinical characteristics and treatment response.^36^ However, no statistically significant correlation between serum bone metabolism markers and cytogenetic abnormalities was found in this study, and the risk stratification based on FISH results did not indicate a significant impact on patient survival. Therefore, we will adopt prospective studies in the future to further expand cases and track changes in serum BMIs after treatment for a long time. Combining clinical features including cytogenetic abnormalities, we will explore their correlation with patient efficacy and prognosis in order to provide more effective information for the diagnosis and treatment of MM.

## Supplementary Material

Supplementary material.docx

## Data Availability

All data used and analyzed are available from the corresponding authors on reasonable request.
