# Supplementary material for: The correlation between serum bone metabolism indexes and bone disease and survival in newly diagnosed multiple myeloma patients
Source: Cancer Biol Ther. 2024 Sep 18;25(1):2403205. doi: 10.1080/15384047.2024.2403205 (PMC11649219; doi:10.1080/15384047.2024.2403205)
Supplement: Supplementary material.docx [file KCBT_A_2403205_SM3567.docx]

Table S 1 Clinical characteristics of 77 MM patients

| Clinical characteristics | N=148 |
| --- | --- |
| Age, median (range) | 64 (57, 67) |
| Male, n (%) | 48 (62.3) |
| ISS, n(%) |  |
| I | 22(28.6) |
| II | 22 (28.6) |
| III | 33 (42.9) |
| M-protein, n (%) |  |
| IgG | 36(46.75) |
| IgA | 19 (24.68) |
| IgD | 1(1.30) |
| Light chains only | 18(23.38) |
| Biclonal subtype | 1(1.30) |
| Non-secretory | 2(2.60) |
| Bone destruction, n (%) | 55(71.43) |
| Serum creatinine (μmol/L), median (Qua) | 83.00 (69.50, 127.25) |
| Serum calcium (mmol/L), median (Qua) | 2.32 (2.20, 2.42) |
| Hemoglobin (g/L), median (Qua) | 100.50 (80.50, 119.00]) |
| Platelet count (10^9/L), median (Qua) | 189.00 (137.00, 273.00) |
| Lactate dehydrogenase (U/L), median (Qua) | 173.50 (136.75, 239.00) |
| β2-microglobulin (mg/L)，median (Qua) | 4500.00 (2290.00, 7401.00) |
| PINP (ng/ml), median (Qua) | 55.34 (38.18, 85.33) |
| β-CTX(μg/ml), median (Qua) | 818.95 (515.30, 1241.50) |
| N-MID (ng/ml), median (Qua) | 19.09 (14.12, 28.33) |

| **A**  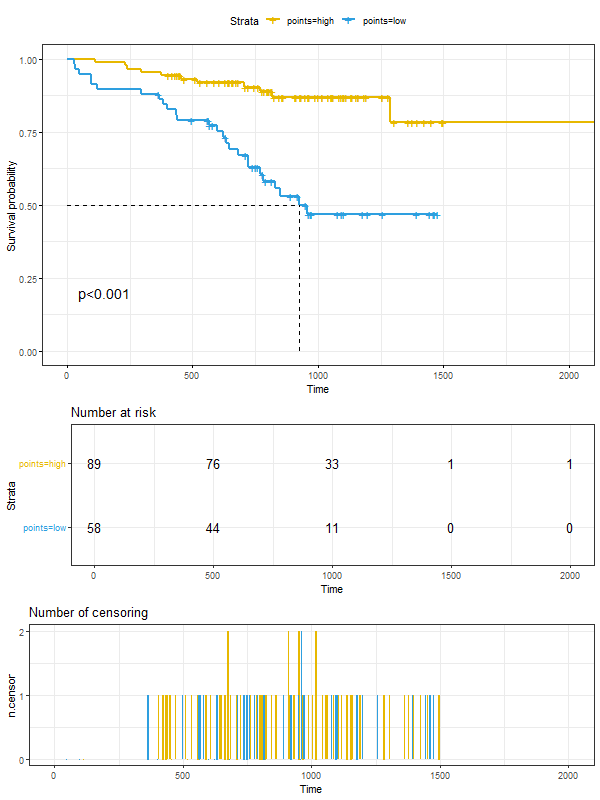 | **B**  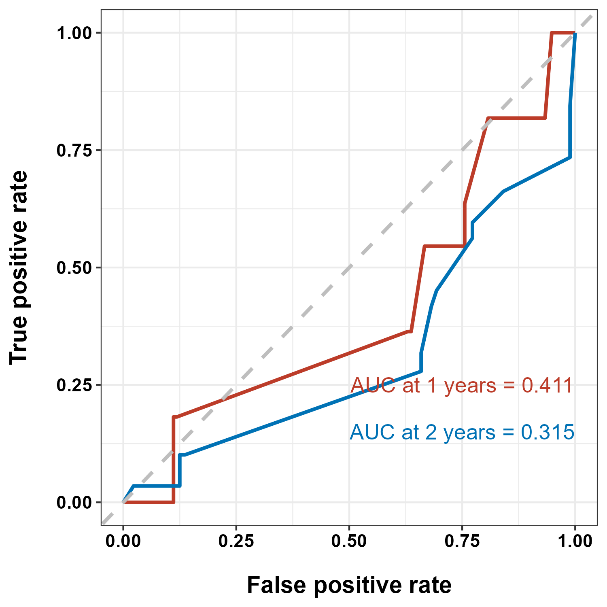 |
| --- | --- |

Figure S 1 Overall survival curves of high/low risk groups according to nomogram(A) and the area under the curve (B).
